# Wide-Gamut and Polarization-Independent Structural Color at Optical Sub-diffraction-Limit Spatial Resolution Based on Uncoupled LSPPs

**DOI:** 10.1186/s11671-019-3050-7

**Published:** 2019-06-25

**Authors:** Jiancun Zhao, Xiaochang Yu, Kui Zhou, Xiaoming Yang, Yiting Yu

**Affiliations:** 1 Key Laboratory of Micro/Nano Systems for Aerospace (Ministry of Education), Northwestern Polytechnical University, Xi’an, 710072 China; 2 Shaanxi Province Key Laboratory of Micro and Nano Electro-Mechanical Systems, Northwestern Polytechnical University, Xi’an, 710072 China

**Keywords:** Structural color, Wide color gamut, Sub-diffraction-limit resolution, Uncoupled LSPP effect, Large viewing angle, Polarization independence

## Abstract

The decreasing pixel size of digital image sensors for high-resolution imaging brings a great challenge for the matching color filters. Currently, the conventional dye color filters with pixel size of several microns set a fundamental limit for the imaging resolution. Here, we put forward a kind of structural color filter with circular nanohole-nanodisk hybrid nanostructure arrays at sub-diffraction-limit spatial resolution based on the uncoupled localized surface plasmon polaritons (LSPPs). Due to the uncoupled LSPPs taking effect, the pixel could generate an individual color even though operating as a single element. The pixel size for the minimum color filtering is as small as 180 × 180 nm^2^, translating into printing pixels at a resolution of ~ 141,000 dots per inch (dpi). In addition, through both the experimental and numerical investigations, the structural color thus generated exhibits wide color gamut, large viewing angle, and polarization independence. These results indicate that the proposed structural color can have enormous potential for diverse applications in nanoscale optical filters, microscale images for security purposes, and high-density optical data storage.

## Introduction

Digital image sensors, which have been widely used for photography, video imaging, and machine vision, are advancing toward the direction of miniaturization and high resolution. It brings a great challenge for the conventional optical elements such as color filters to improve the spatial resolution [1]. An ultrahigh-resolution digital image sensor with imaging unit size of 50 nm by vertical nanorod arrays was demonstrated in 2015 [2], while the unit size of traditional color filter mainly fabricated by organic dye polymers or chemical pigments was as large as several micrometers. Thus, one color filter unit will cover several imaging units and cause a loss to the imaging resolution, which could not meet the demand for the future high-resolution imaging [3].

Recently, color filtering based on structural colors provides an alternative method to control light spatially [4–6]. The structural color is mainly based on the interaction between light and various nanostructures rather than materials, so it is capable to generate much smaller pixel sizes than the pixels achieved today in image sensors [7–11]. Abbe’s classical diffraction limit states that the minimum resolvable distance between two closely spaced objects is at best half the wavelength used for imaging in visible light [12]. Since the discovery of extraordinary optical transmission (EOT) phenomenon in 1998 [13], plasmonic effects have been widely used for designing structural color filters (SCFs), providing a possibility for the color filter to realize a spatial resolution reaching the sub-diffraction limit [14–17]. At present, many kinds of SCFs have been reported with a variety of plasmonic nanostructures [18], such as periodic subwavelength nanohole arrays [19–21], plasmonic nanodisks [22–24], hybrid nanohole-nanodisk structures [25–28], and subwavelength metal gratings [29–32]. For the applications of SCFs in image sensors, the small pixel size, wide color gamut, large viewing angle, and polarization independence are the major issues to be addressed. Burgos et al. exhibited a kind of plasmonic SCFs based on the periodic metallic subwavelength hole arrays. The colors were set by the periodicity of plasmonic building blocks due to the coupling effect, resulting in micrometer-sized pixels [33]. Structural colors generated from all-dielectric metasurfaces with a high refractive index and low loss offer high saturation and high efficiency [34, 35]. Sun et al. presented a kind of all-dielectric structural color generated by the electric and magnetic resonances in TiO_2_ metasurfaces. However, the distinct colors could only be observed when the metasurface was reduced to around 1.6 μm [36]. Horie et al. reported a kind of transmissive color filters based on periodical subwavelength silicon nanoholes that could replace conventional dye-based color filters used in backside-illuminated CMOS image sensor technologies. Nevertheless, its pixel size could only be shrinked down to nearly 1 μm and only had an insensitive response to *a* ± 20° angular range [37]. Yang et al. introduced a kind of reflective color filter based on asymmetric Fabry-Perot cavities, which could get a minimum pixel size of 500 nm [38]. Zeng et al. demonstrated a kind of plasmonic subtractive color filter based on the one dimensional (1D) nanogratings patterned in a single optically thin Ag film, generating extremely small pixel size close to the optical diffraction limit due to the short-range interactions of surface plasmon polaritons (SPPs). However, it was sensitive to the incident polarization [39]. Kumar et al. presented an approach for full-color printing by encoding color information into Ag/Au nanodisks raised above a holey backreflector. The color thus generated was preserved even as individual pixels of 250 × 250 nm^2^, enabling color printing at a resolution of ~ 100,000 dpi, closing to the diffraction-limited resolution [40]. Small (tens of nanometers) isolated semiconductor nanostructures can be used to generate the scattering colors; however, they do not scatter strongly enough to be viewed plainly in a bright-field reflection microscope [41].

Here, we propose a kind of structural color with circular nanohole-nanodisk hybrid nanostructure arrays based on the uncoupled localized surface plasmon polaritons (LSPPs), obtaining an individual color pixel size of 180 × 180 nm^2^, corresponding to a spatial resolution of ~ 141, 000 dpi. In addition, the structural color thus generated reveals a wide color gamut with a large viewing angle and strong polarization insensitive property. An illustrative color palette is obtained by changing the geometrical parameters of the hybrid nanostructures, including the primary component colors of cyan, magenta, and yellow (CMY). The simulation results demonstrate that the realized colors exhibit a large angular invariant feature up to ± 40°. Moreover, the circular shape of nanostructures makes the demonstrated structural color reveal a strong polarization independence. Furthermore, due to the uncoupled LSPPs taking effect in light field modulation, the individual color pixel can be generated even though operating as a single element, resulting in the achievement of sub-diffraction-limit resolution. As a proof-of-concept demonstration, an image containing colorful letters is printed by the suggested nanostructures.

## Methods

**Fig. 1:**
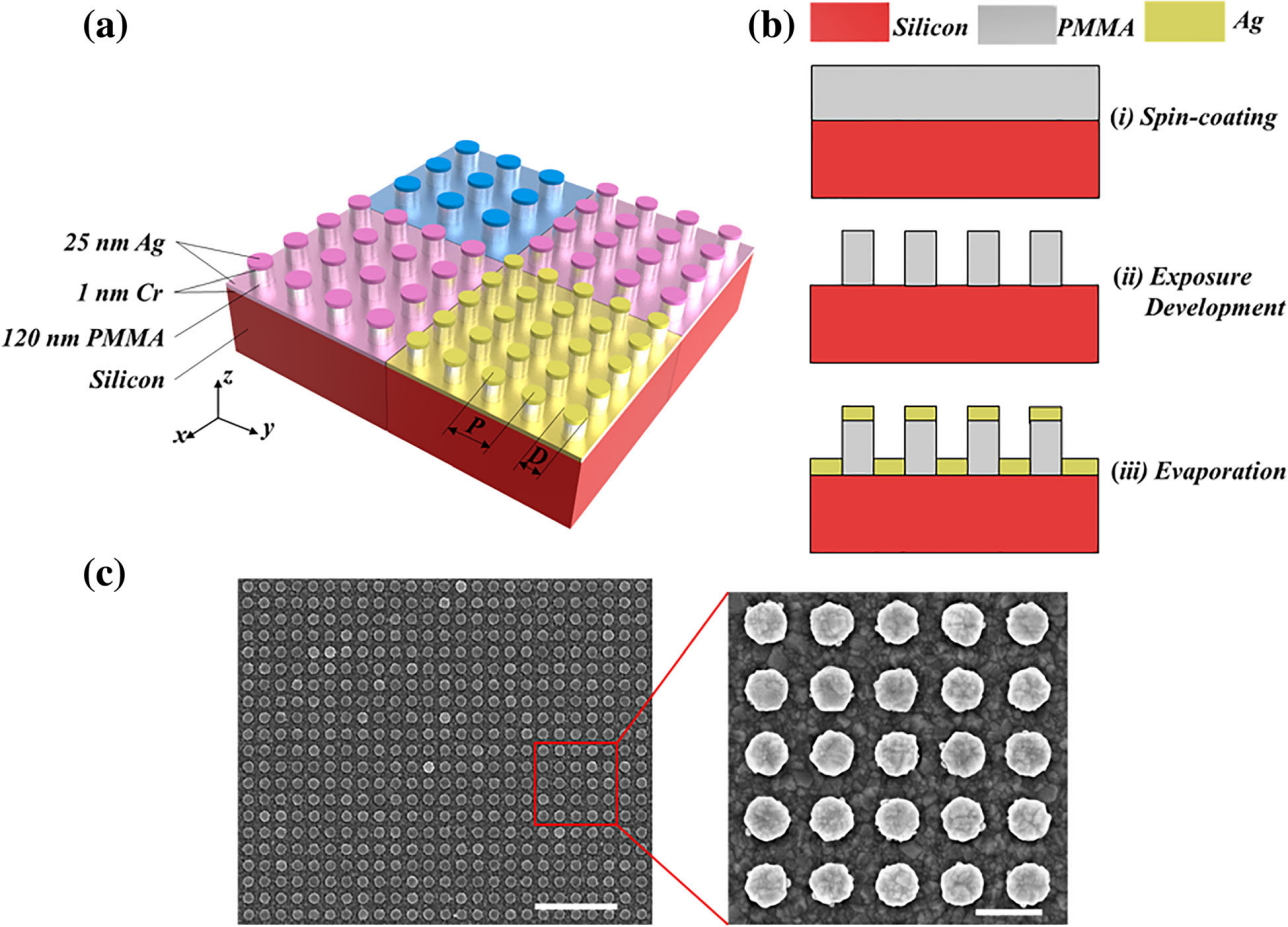
**a** Illustration of the circular nanodisk-nanohole hybrid nanostructure arrays on silicon substrate. **b** The schematics of the fabrication process for the designed nanostructures. **c** SEM images of the fabricated nanostructure arrays with *P* = 200 nm and *D* = 130 nm. The inset gives an enlarged view. The scale bars are 1 μm (left) and 200 nm (right)

The proposed plasmonic structural colors are reflective square-lattice circular nanodisk-nanohole hybrid nanostructure arrays on silicon substrate, as shown in Fig. 1a. The 25 nm Ag was directly evaporated onto the 120 nm polymethyl methacrylate (PMMA) pillars with 1 nm Cr as the adhesion layer. Here, silicon was selected as the substrate due to its high conductivity, which is convenient for the electron-beam lithography (EBL) fabrication. Ag was specifically chosen as the metallic layer due to its low extinction coefficient. Furthermore, its inherent formation of a thin (~ 2–3 nm) oxide layer (Ag_2_O) that will cause a slight shift in the spectra, but it has a little effect on the structural color performance [17].

Figure 1b shows the schematics of the fabrication process for the nanostructures as suggested. Firstly, the electron-beam resist PMMA with the thickness of 120 nm was spin-coated onto the silicon substrate (Fig. 1b-i). And then, the PMMA nanopillar templates were exposed by the NanoBeam Limited nB5 system with an accelerating voltage of 100 kV and a beam current of 100 pA. The development process was performed by immersing the sample in methyl isobutyl ketone (MIBK) solution at 25 °C for 2 min, followed by rinsing in isopropyl alcohol (IPA) for 2 min. Finally, the sample was blow-dried under a steady stream of N_2_ (Fig. 1b-ii). And then, an adhesion layer of Cr (1 nm) and an Ag film (25 nm) were deposited by an e-beam evaporator system (Fig. 1b-iii). Figure 1c shows the SEM images of the ultimately achieved circular nanodisk-nanohole hybrid structure array.

## Results and Discussion

### Wide Color Gamut

**Fig. 2:**
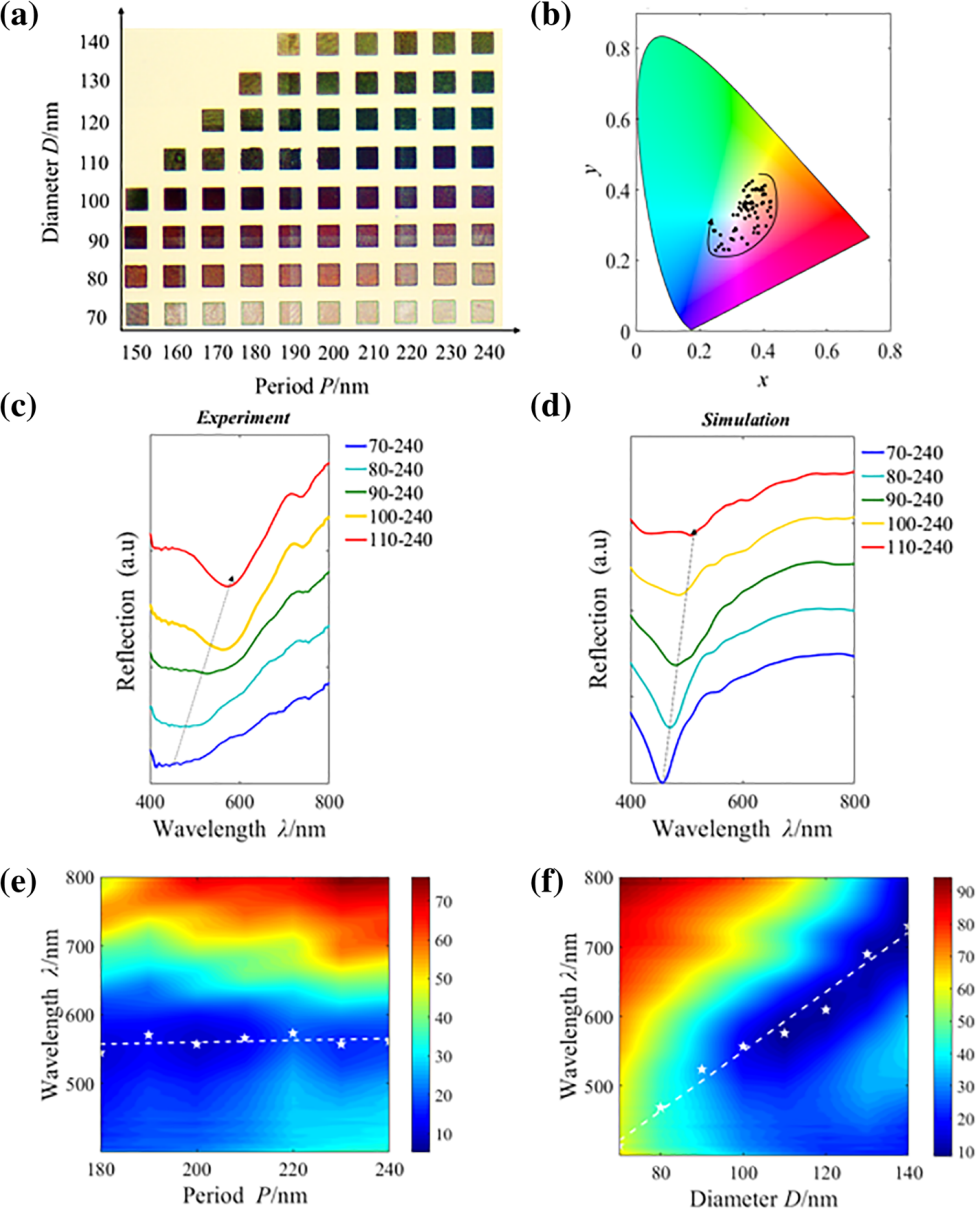
**a** Recorded color palette of the reflective subtractive colors as functions of the period *P* (varying from 150 to 240 nm in an increment of 10 nm) and diameter *D* (varying from 70 to 140 nm in an increment of 10 nm). Each palette square has a size of 8 × 8 μm^2^, and the whole array is under illumination by an un-polarized white light. **b** CIE1931 chromaticity diagram overlaid with the black points corresponding to the colors extracted from **a**. Experimental (**c**) and simulated (**d**) reflective spectra of the nanostructure arrays with different geometrical parameters. For example, “70–240” means *D* = 70 nm, *P* = 240 nm. **e** Contour map of the experimental reflective spectra as a function of the incident wavelength and period. The period *P* changes from 180 to 240 nm, while keeping *D* = 100 nm as a constant. **f** Experimentally reflection contour map for nanostructure arrays with different diameters changing from 70 to 140 nm at a constant period of 230 nm. The white asterisks represent the valleys’ positions (*λ*_min_), and the white dashed lines refer to the fitted straight lines with the corresponding valleys

Figure 2a displays a palette of experimentally reflected colors obtained by changing the diameter *D* and period *P* of the nanostructure arrays. Corresponding positions of these colors are plotted in the CIE 1931 color space, as shown in Fig. 2b, which confirms the capability for achieving the main CMY colors ranging from cyan to magenta to yellow. The reflectivity is then characterized using the NOVA-EX spectrometer established on the microscopic system (Olympus-BX53) with the illuminating wavelength ranging from 400 to 800 nm. The reflection signals are collected by an objective lens (MPlanFL N, NA = 0.9, 100×). Figure 2c presents the experimental reflective spectra of the samples, the valleys redshift as *D* varies from 70 to 110 nm. Moreover, for the same structures, the simulated reflective spectra obtained by the finite-difference time-domain (FDTD) method shown in Fig. 2d are in qualitative agreement with the corresponding experimental results, where valleys redshift with the increasing *D*. However, it still exists a little difference due to the shape and size deviations from nanofabrication, and the refractive indices, as well as the thicknesses in the experiment, could be slightly different from those used in the simulation. The contour maps of the experimental reflective spectra plotted in Fig. 2e, f demonstrate that the impact of period *P* on spectral modulation is fairly small, while the diameter *D* plays a dominant role for the spectral control, which is different from the situation where the period is the main factor reported in other common literatures [19–21, 33, 36, 37]. And this property makes it possible to define colors with only one single nanostructure.

### Physical Mechanism

It is known that the optical properties of periodical nanostructures are largely dependent on the distance between nanostructures, especially when the distance is relatively small. This is because the coupling effect associated with the hybridization of the dipole or higher multipolar plasmons between nanostructures lead to variations in the collective plasmon energy [26, 42, 43]. However, the coupling effect limits the pixel size, and sometimes causes the non-negligible resonant peak shift or peak split, thus leading to unexpected color generation [17]. Due to the short propagation distance of short-range surface plasmon polaritons (SRSPPs) and small decay length of LSPPs, as the separation increases, the coupling effect becomes weaker, and interactions between neighboring nanostructures become negligible [23]. Hence, in order to avoid the coupling effect and achieve a kind of structural color reached to the sub-diffraction-limit resolution, the space between nanoparticles must be large enough and the size of the unit cell should be less than the diffraction-limited size.

**Fig. 3:**
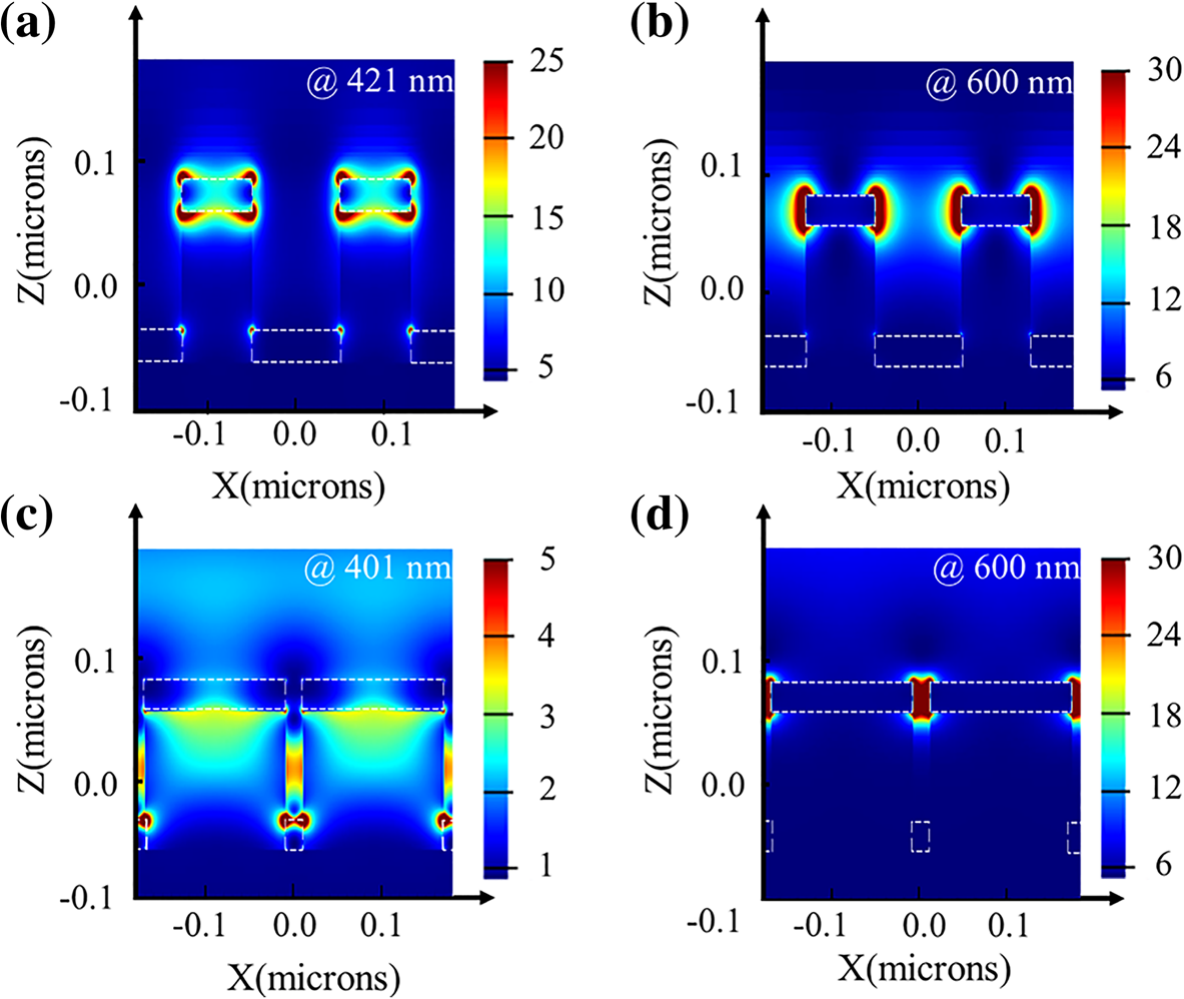
Distribution of the electric field (|*E|*^2^) in the *XZ* plane for the structure with **a**, **b** *D* = 80 nm, *P* = 180 nm, and **c**, **d** *D* = 160 nm, *P* = 180 nm. **a**, **c** Illuminated at the reflection valleys. **b**, **d** Both illuminated at the long incident wavelength of 600 nm. The white dashed lines are the boundaries of the Ag layer

In order to analyze the underlying physical mechanism of the color filtering effect, the nanostructure arrays with large and small inter-particle distances have been analyzed by using the FDTD method. Figure 3 presents the simulated electric field (*|E|*^2^) distribution results at reflective valleys and long incident wavelength of 600 nm, respectively. For the structure with a large inter-particle distance, no matter at short (Fig. 3a) or long (Fig. 3b) incident wavelength, the strong electric-field intensity distributions are both merely confined at the edges of the nanodisks and nanoholes, demonstrating that there is nearly no coupling LSPPs existing. In comparison, for the structure with a small inter-particle distance, as shown in Fig. 3c, the electric-field intensity confined on the Ag/Air interface demonstrates that it exists the SRSPPs coupling effect at short incident wavelength. And in Fig. 3d, the electric-field intensity limited in the gap between nanodisks illustrates that there is a strong LSPPs coupling effect at long incident wavelength. Therefore, when the distance is small, both the LSPPs and the SRSPPs coupling effect are in charge of light field modulation, while for the structure with a larger distance, there is nearly no coupling effect.

In our design, the inter-particle distance is large enough to avoid the coupling effect, so the observed colors in Fig. 2a are mainly modulated by the uncoupled LSPP modes. The property of LSPP mode is relevant to the shape and size of the nanoparticles [44–46]; thus, the resonant wavelength of the designed structure is mainly controlled by the diameters of the nanostructure (shown in Fig. 2f). And due to the uncoupling effects, the reflective valleys stay almost unchanged as the period increases, corresponding with the experimental results shown in Fig. 2e.

### Polarization Independence and Large Viewing Angle

**Fig. 4:**
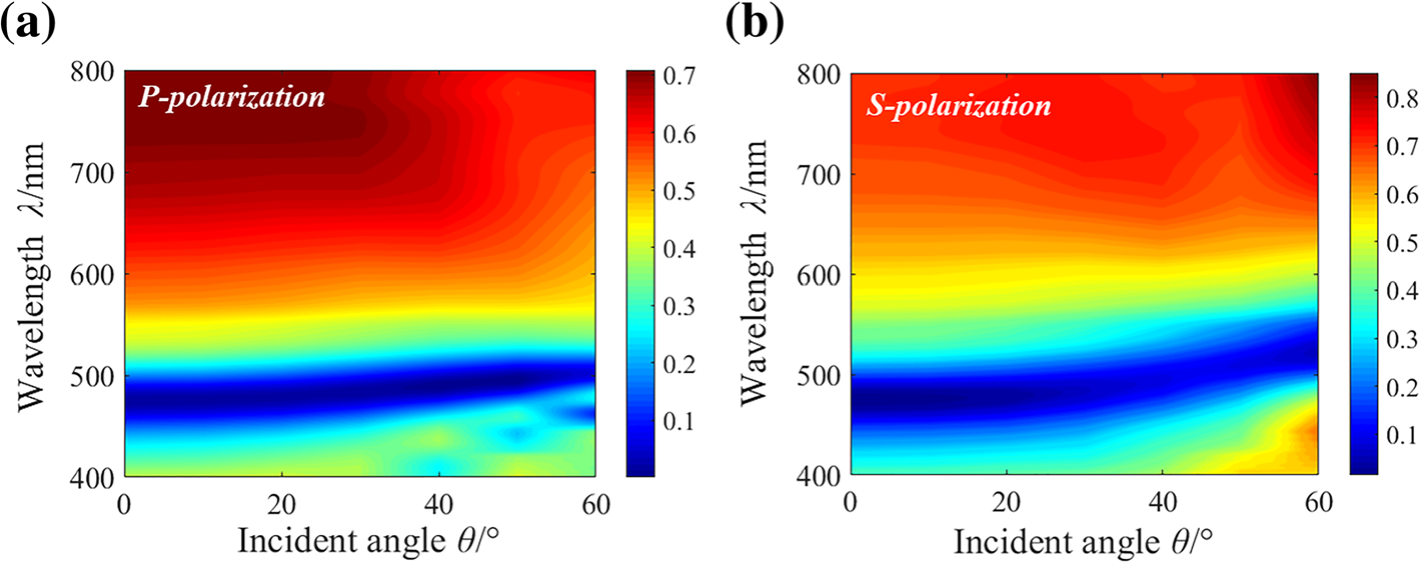
Contour map of the simulated angle-resolved reflectance spectra for the structure with *P* = 180 nm, *D* = 80 nm under **a** *p*-polarized and **b** *s*-polarized illumination

Both polarization independence and large viewing angle are necessary for the color filter in image sensing applications. Considering the circular shape of the nanostructure is symmetric along the *x* and *y* directions, it can be concluded that the proposed structural color is polarization independent. To investigate the viewing angle effect, the reflective spectra under various incident light angles have been analyzed by the FDTD method. The simulation model is built based on the schematic diagram shown in Fig. 1a. And the Broadband Fixed Angle Source Technique (BFAST) is used. The complex refractive indices of the material for simulations are based on the data from Palik in the material library of the software. The simulated results for both *p*-polarization and *s*-polarization shown in Fig. 4a, b illustrate that the reflective spectra almost keep invariant with the incident angle up to ± 40°, demonstrating a large viewing angle.

### Super High Resolution

**Fig. 5:**
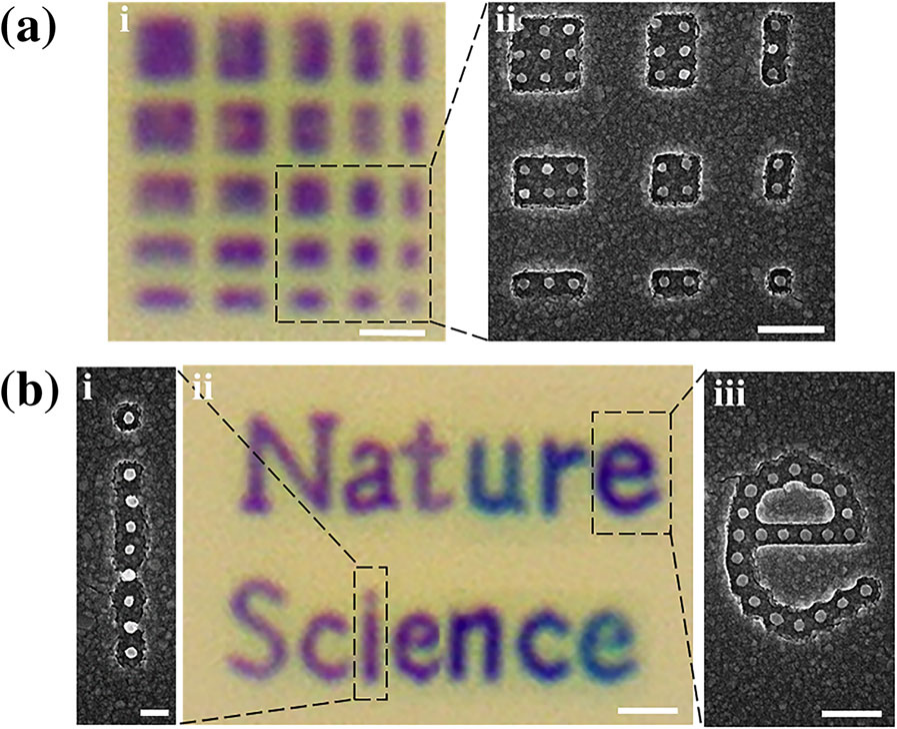
**a** Color printing resolution test pattern. **b** Subwavelength color printing of “Nature” and “Science” with the size of 6 μm × 9 μm. The scale bars are **a**-i 1 μm, **a**-ii 500 nm, **b**-i 200 nm, **b**-ii 1 μm, and **b**-iii 500 nm

Owing to the uncoupled LSPPs, our design offers a kind of high spatial resolution structural color with pixel size at optical sub-diffraction limit. To verify the achievement of super high resolution, a set of resolution test structures are fabricated. The checkered patterns consisting of nanostructures with 5 × 5, 5 × 4, … , 2 × 1, 1 × 1 arrays with size of *P* = 180 nm, and *D* = 80 nm are shown in Fig. 5a (a bright-field microscope optical image (left) and an SEM image (right)). As expected, in Fig. 5a-i, the arrays with only one nanostructure can still generate the magenta color, even though it is a single pixel without periodicity. The individual magenta pixel with a unit cell area of 180 × 180 nm^2^ demonstrates that this structure could form a pixel of color on a 180-nm-pitch grid and reach to a super high resolution of ∼ 141,000 dpi.

The designed color pixels for subwavelength printing applications are demonstrated by showing microscopic colorful letters with sub-diffraction-limit pixel resolution. We printed the letters of “Nature, Science” with the corresponding structural colors, as shown in Fig. 5b-ii. Figure 5b-i, b-iii shows the SEM images of the regions outlined in Fig. 5b-ii. In Fig. 5b-ii, the top point on the letter “i” can be clearly visible, once again proving that even a single nanostructure can act as a color element. This feature gives rise to print resolution at the single-nanostructure level, which could provide extremely high spatial resolution for applications in high-density optical data storage and microscale images for security purposes.

## Conclusions

In conclusion, the structural color at optical sub-diffraction-limit spatial resolution generated by the circular nanohole-nanodisk hybrid structure arrays is introduced, which exhibits the wide color gamut, large viewing angle, and strong polarization independence. Due to the uncoupled LSPPs taking effect, the color pixel sizes can reach to 180 × 180 nm^2^, exhibiting a high resolution up to ~ 141,000 dpi. And by simply changing the geometrical parameters of the nanostructure, the demonstrated structural color can span the whole CMY color system. Moreover, the simulation results demonstrate that the structural color exhibits a high angular tolerance up to ± 40°. Furthermore, this structure has the advantage of individual color generation at a sub-diffraction-limit pixel. As a proof-of-concept demonstration, a colorful letter image has been acquired with this structure. The proposed plasmonic structural color thus generated has the potential for applications in nanoscale color filters to satisfy the demand about super-high-resolution imaging, and could be used for security purposes, and high-density optical data storage.

## Data Availability

The datasets supporting the conclusions of this article are included within the article.

## Abbreviations

BFAST: Broadband Fixed Angle Source Technique
CMY: Cyan, magenta, yellow
dpi: Dots per inch
EBL: Electron-beam lithography
EOT: Extraordinary optical transmission
FDTD: Finite-difference time-domain
IPA: Isopropyl alcohol
LSPPs: Localized surface plasmon polaritons
MIBK: Methyl isobutyl ketone
PMMA: Polymethyl methacrylate
SCFs: Structural color filters
SPPs: Surface plasmon polaritons
SRSPPs: Short-range surface plasmon polaritons

## References

[1] Chen Q, Hu X, Wen L, Yu Y, Cumming DRS (2016). Nanophotonic image sensors. Small.

[2] Jiang C, Song J (2015). An ultrahigh-resolution digital image sensor with pixel size of 50 nm by vertical nanorod arrays. Adv. Mater.

[3] Catrysse PB, Wandell BA (2005). Roadmap for CMOS image sensors: Moore meets Planck and Sommerfeld. Proc. SPIE - IS &T.

[4] Chen Q, Das D, Chitnis D, Walls K, Drysdale TD, Collins S, Cumming DRS (2012). A CMOS image sensor integrated with plasmonic colour filters. Plasmonics.

[5] Nagasaki Y, Suzuki M, Takahara J (2017). All-dielectric dual-color pixel with subwavelength resolution. Nano Lett.

[6] Frey L, Parrein P, Raby J, Pellé C, Hérault D, Marty M, Michailos J (2011). Color filters including infrared cut-off integrated on CMOS image sensor. Opt Express.

[7] Dumanli AG, Savin T (2016). Recent advances in the biomimicry of structural colours. Chem Soc Rev.

[8] Roberts AS, Pors A, Albrektsen O, Bozhevolnyi SI (2014). Subwavelength plasmonic color printing protected for ambient use. Nano Lett.

[9] Shrestha VR, Lee SS, Kim ES, Choi DY (2014). Aluminum plasmonics based highly transmissive polarization-independent subtractive color filters exploiting a nanopatch array. Nano Lett.

[10] Yokogawa S, Burgos SP, Atwater HA (2012). Plasmonic color filters for CMOS image sensor applications. Nano Lett.

[11] Williams C, Rughoobur G, Flewitt AJ, Wilkinson TD (2017). Nanostructured plasmonic metapixels. Sci Rep.

[12] Abbe E (1874). A contribution to the theory of the microscope and the nature of microscopic vision. Proc Bristol Nat Soc.

[13] Ebbesen TW, Lezec HJ, Ghaemi HF, Thio T, Wolff PA (1998). Extraordinary optical transmission through sub-wavelength hole arrays. Nature.

[14] Miyata M, Hatada H, Takahara J (2016). Full-color subwavelength printing with gap-plasmonic optical antennas. Nano Lett.

[15] Zhu X, Vannahme C, HøjlundNielsen E, Mortensen NA, Kristensen A (2016). Plasmonic colour laser printing. Nat Nanotechnol.

[16] Rajasekharan R, Balaur E, Minovich A, Collins S, James TD, Djalalian-Assl A, Ganesan K, Tomljenovic-Hanic S, Kandasamy S, Skafidas E, Neshev DN, Mulvaney P, Roberts A, Prawer S (2013). Filling schemes at submicron scale: development of submicron sized plasmonic colour filters. Sci Rep.

[17] Lu BR, Xu C, Liao J, Liu J, Chen Y (2016). High-resolution plasmonic structural colors from nanohole arrays with bottom metal disks. Opt Lett.

[18] Yu P, Besteiro LV, Huang Y, Wu J, Fu L, Tan HH, Jagadish C, Wiederrecht GP, Govorov AO, Wang Z (2019). Broadband metamaterial absorbers. Adv Opt Mater.

[19] Zhao J, Gao B, Li H, Yu X, Yang X, Yu Y (2017). Biomimetic plasmonic color generated by the single-layer coaxial honeycomb nanostructure arrays. J Nanophoton.

[20] Li Y, Yue WJ, Chen ZX, Cao BQ, Fu XQ, Zhang CW, Li ZM (2018). Large-area structural color filtering capitalizing on nanoporous metal- dielectric-metal configuration. Nanoscale Res Lett.

[21] Inoue D, Miura A, Nomura T, Fujikawa H, Sato K, Ikeda N, Tsuya D, Sugimoto Y, Koide Y (2011). Polarization independent visible color filter comprising an aluminum film with surface-plasmon enhanced transmission through a subwavelength array of holes. Appl Phys Lett.

[22] Yang C, Hong L, Shen W, Zhang Y, Liu X, Zhen H (2013). Design of reflective color filters with high angular tolerance by particle swarm optimization method. Opt Express.

[23] Ye M, Sun L, Hu X, Shi B, Zeng B, Wang L, Zhao J, Yang S, Tai R, Fecht HJ, Jiang JZ, Zhang DX (2015). Angle-insensitive plasmonic color filters with randomly distributed silver nanodisks. Opt Lett.

[24] Si G, Zhao Y, Lv J, Lu M, Wang F, Liu H, Xiang N, Huang TJ, Danner AJ, Teng J, Liu YJ (2013). Reflective plasmonic color filters based on lithographically patterned silver nanorod arrays. Nanoscale.

[25] Zhao J, Yu X, Yang X, Xiang Q, Duan H, Yu Y (2017). Polarization independent subtractive color printing based on ultrathin hexagonal nanodisk-nanohole hybrid structure arrays. Opt Express.

[26] Wu QJ, Jia H, Hu XL, Sun LB, Wang LS, Yang SM, Tai RZ, Fecht HJ, Wang LQ, Zhang DX, Jiang JZ (2017). Plasmonic reflection color filters with metallic random nanostructures. Nanotechnol.

[27] Mudachathi R, Tanaka T (2017). Up scalable full colour plasmonic pixels with controllable hue, brightness and saturation. Sci Rep.

[28] Zhao J, Yu X, Yang X, Tee CA, Yuan W, Yu Y (2019). Polarization-independent and high-efficiency broadband optical absorber in visible light based on nanostructured germanium arrays. Opt Lett.

[29] Koirala I, Shrestha VR, Park CS, Lee SS, Choi DY (2017). Polarization-controlled broad color palette based on an ultrathin one-dimensional resonant grating structure. Sci Rep.

[30] Kaplan AF, Xu T, Guo LJ (2011). High efficiency resonance-based spectrum filters with tunable transmission bandwidth fabricated using nanoimprint lithography. Appl Phys Lett.

[31] Park CH, Yoon YT, Shrestha VR, Park CS, Lee SS, Kim ES (2013). Electrically tunable color filter based on a polarization-tailored nano-photonic dichroic resonator featuring an asymmetric subwavelength grating. Opt Express.

[32] Shen C, Diaz-Rubio A, Li J, Cummer SA (2018). A surface impedance-based three-channel acoustic metasurface retroreflector. Appl Phys Lett.

[33] Burgos SP, Yokogawa S, Atwater HA (2013). Color imaging via nearest neighbor hole coupling in plasmonic color filters integrated onto a complementary metal-oxide semiconductor image sensor. ACS Nano.

[34] Yang B, Liu W, Li Z, Cheng H, Chen S, Tian J (2018). Polarization-sensitive structural colors with hue-and-saturation tuning based on all-dielectric nanopixels. Adv Optical Mater..

[35] Li J, Liu C, Wu T, Liu Y, Wang Y, Yu Z, Ye H, Yu L (2019). Efficient polarization beam splitter based on all-dielectric metasurface in visible region. Nanoscale Res Lett.

[36] Sun S, Zhou Z, Zhang C, Gao Y, Duan Z, Xiao S, Song Q (2017). All-dielectric full-color printing with TiO_2_ metasurfaces. ACS Nano.

[37] Horie Y, Han S, Lee JY, Kim J, Kim Y, Arbabi A, Shin C, Shi L, Arbabi E, Kamali SM, Lee HS, Hwang S, Faraon A (2017). Visible wavelength color filters using dielectric subwavelength gratings for backside-illuminated CMOS image sensor technologies. Nano Lett.

[38] Yang Z, Zhou Y, Chen Y, Wang Y, Dai P, Zhang Z, Duan H (2016). Reflective color filters and monolithic color printing based on asymmetric Fabry-Perot cavities using nickel as a broadband absorber. Adv Opt Mater.

[39] Zeng B, Gao Y, Bartoli FJ (2013). Ultrathin nanostructured metals for highly transmissive plasmonic subtractive color filters. Sci Rep.

[40] Kumar K, Duan H, Hegde RS, Koh SCW, Wei JN, Yang JKW (2012). Printing colour at the optical diffraction limit. Nat Nanotechnol.

[41] Kuznetsov AI, Miroshnichenko AE, Brongersma ML, Kivshar YS, Luk’yanchuk B (2016). Optically resonant dielectric nanostructures. Science.

[42] Offermans P, Schaafsma MC, Rodriguez SRK, Zhang Y, Crego-Calama M, Brongersma SH, Rivas JG (2011). Universal scaling of the figure of merit of plasmonic sensors. ACS Nano.

[43] Rajeeva BB, Lin L, Zheng Y (2018). Design and applications of lattice plasmon resonances. Nano Res.

[44] Willets KA, Duyne RPV (2007). Localized surface plasmon resonance spectroscopy and sensing. Annu Rev Phys Chem.

[45] Barnes WL, Dereux A, Ebbesen TW (2003). Surface plasmon subwavelength optics. Nature.

[46] Genet C, Ebbesen TW (2007). Light in tiny holes. Nature.

